# Recent trends in the incidence of hip fracture in Tottori Prefecture, Japan: changes over 32 years

**DOI:** 10.1007/s11657-020-00823-3

**Published:** 2020-10-02

**Authors:** Hiroshi Hagino, Mari Osaki, Reiko Okuda, Shinpei Enokida, Hideki Nagashima

**Affiliations:** 1 Rehabilitation Division, Tottori University Hospital, Yonago, Japan; 2 School of Health Science, Faculty of Medicine, Tottori University, Yonago, 683-8503 Japan; 3 Department of Orthopedic Surgery, Faculty of Medicine, Tottori University, Yonago, Japan

**Keywords:** Hip fracture, Epidemiology, Neck fracture, Trochanteric fracture

## Abstract

**Summary:**

The incidence rate of hip fracture in Tottori Prefecture tended to increase until 2018 in men, but it did not increase after 2010 in women. By type of fracture, the incidence rate of femoral neck fractures also increased over time in men, but no other changes were observed from 2010.

**Purpose:**

The aims of this study were to determine the sex-, age-, and fracture-type-specific incidence rates of hip fractures in Tottori Prefecture between 2007 and 2018 and to compare the results with our past results to identify changes over time.

**Methods:**

All hip fractures in people aged 35 years or older living in Tottori Prefecture were surveyed from 2007 to 2018 throughout the entire prefecture, and the age- and sex-specific incidence rates were calculated. The incidence rates from 1986 to 1988, 1992 to 1994, 1998 to 2000, and 2004 to 2006 previously reported were used for the analysis.

**Results:**

In men, the age-adjusted number of patients adjusted by demographic structure based on the mean incidence rate for each 3-year period from 1986 to 2018 showed an increase in incidence over time compared with the incidence for 1986–1988 (*p* < 0.001). In women, the incidence rose over time compared with the incidence for 1986–1988 until 2004–2006 (*p* < 0.001), and no further increase was observed from 2010. The age-specific incidence rates of neck fracture in men were higher in 2010–2012 and 2016–2018 compared with 2004–2006 (*p* < 0.001), but those in women showed no increase with time. Those of trochanteric fracture did not change over time in either men or women.

**Conclusion:**

The hip fracture incidence rate in Tottori Prefecture, Japan, tended to increase until 2018 in men, but it did not increase after 2010 in women.

**Electronic supplementary material:**

The online version of this article (10.1007/s11657-020-00823-3) contains supplementary material, which is available to authorized users.

## Introduction

Hip fractures are quite common among older adults and cause a significant decline in mobility after the fracture and decreased life expectancy. Almost all cases require surgery, and decline in mobility leads to the need for nursing care, resulting in tremendous nursing care costs. For this reason, the condition features prominently in healthcare and has a significant socioeconomic impact. Determining changes in incidence rates is therefore extremely useful for future implementation of healthcare policies. Hip fractures are divided into neck fractures and trochanteric fractures, which differ in frequency of occurrence by age, clinical condition, and treatment method. Trochanteric fractures have a stronger association with vertebral fractures than neck fractures, and the clinical condition has been shown to differ between the two types [1]. However, few studies have examined the incidence rate and changes over time for the two types of fractures.

Japan is aging at the fastest rate in the world. In 2018, 28.1% of the country’s population was at age 65 years or older, and 4.5% was 85 years or older [2]. Since 1986, we have been surveying all hip fracture cases in Tottori Prefecture in Japan, and we have reported the sex- and age-specific incidence rates [3–5]. From 1986 to 2006, we observed both an increase in the number of fractures and a significant rise in age group–specific incidence rates over time for men and women. In a study of sex- and age group–specific incidence rates of neck fractures and trochanteric fractures separately, we found that the changes with age differed between the two types of fractures.

We then continued to survey all hip fracture cases in Tottori Prefecture until 2018. In the present study, the aims were to determine the number of hip fracture cases and the sex-, age-, and fracture-type-specific incidence rates in Tottori Prefecture between 2007 and 2018 and to compare the results with our past results to determine changes over time.

## Patients and methods

### Registration of fracture patients

A survey of all cases of hip fracture was performed, similar to our previous study [4], covering all of Tottori Prefecture for every year from 2007 to 2018, except for 2015. The population in Tottori Prefecture was 599,830 people in 2007 (286,337 men; 313,493 women) and 560,517 people in 2018 (267,885 men; 292,632 women), showing a decrease over time. The size of the over-35 population did not change, with 386,102 people in 2007 (176,780 men; 209,322 women) and 386,940 people in 2018 (178,594 men; 208,346 women). Those aged 65 years or older accounted for 25.1% of the population in 2007 and 31.3% in 2018, while those aged 85 years or older accounted for 3.8% and 6.3%, respectively, and those aged 90 years or older accounted for 1.5% and 2.7%, respectively, showing an increase in the proportion of the oldest old in the population over time.

We used the same methods [3, 4] as in our previous studies to survey the patients. Specifically, all hip fractures in patients aged 35 years or older that occurred between 2007 and 2018 (except 2015) were examined in all hospitals in Tottori Prefecture. This included 36 hospitals, and according to the hospital records, survey registration was performed by the doctors or medical staff in each of these hospitals. Registration information included sex, age, area of residence, date of fracture, type of fracture (neck or trochanteric), and fracture location (indoors or outdoors). Patients residing in other prefectures were excluded. Duplication of cases was determined using the patients’ ages, dates of fracture, types of fracture, and areas of residence. The data collection methods at the two or three hospitals were investigated each year, as previously reported [3, 4], and this confirmed that the methods used to register the patients with hip fractures were consistent.

### Statistical analysis

The patients were divided into groups by age (subdivided into 5-year increments), sex, and fracture type (neck or trochanteric fracture). The age- and sex-specific incidence rates (per 100,000 person-years) were calculated based on the population of the Tottori prefecture each year. Every 5 years in Japan, a national census is performed in October 1, including 2010 and 2015 during the observation period. The age- and sex-specific populations for each survey year were estimated by the Bureau of Statistics of the Tottori Prefecture Government Office according to resident registration records, which are updated each time when residents change their address and submit notices of the changes (https://www.pref.tottori.lg.jp/275032.htm).

The age- and sex-specific incidence rates (per 100,000 person-years) from 1986 to 1988, 1992 to 1994, 1998 to 2000, and 2004 to 2006, which we previously reported, were used for this analysis [3–5]. The expected number of patients, age-adjusted to the population structure from 1986 in all of Japan (35 years and older), was calculated from the age- and sex-specific incidence rates in each observation year. The average change in the fracture rate per 100,000 per year was estimated using regression coefficients in a linear regression model, with incidence rate as the dependent variable and year as the independent variable. The monthly variation in the number of patients was tested by Friedman’s test. Statistical analysis was conducted using SPSS software for Windows, version 24 (IBM, Armonk, NY, USA), with a significance level of 5%.

## Results

### Characteristics of patients aged 35 years and older with hip fractures

An increase was observed in the total number of men with hip fractures during the observation period from 2007 to 2018, from 163 men to 304 men, whereas the number of women with hip fractures increased from 675 in 2007 to 1015 in 2013 and then plateaued (Table 1). The number of female patients was 3.1 to 4.5 times higher than the number of male patients. In men, the age group–specific number of patients was the highest in the 85–89-year-old age group, except from 2010 to 2012, when it was the highest in the 80–84-year-old age group. In women, the highest was in the 90 years and older age group, except in 2007 and 2010, when it was the highest in the 85–89-year-old age group (Supplement 1).

**Table 1:** Number of patients

Year	Total		Fracture type					
			Neck		Trochanteric		Undetermined	
	Men	Women	Men	Women	Men	Women	Men	Women
2007	163	675	71	289	90	384	2	2
2008	195	806	64	317	129	488	2	1
2009	202	690	78	288	121	400	3	2
2010	197	892	87	377	109	508	1	7
2011	249	842	102	352	147	490	0	0
2012	233	944	116	390	117	554	0	0
2013	248	1015	111	442	136	572	1	1
2014	242	876	126	379	113	481	3	16
2016	281	943	149	379	131	561	1	3
2017	269	953	155	407	113	544	1	2
2018	304	956	173	427	131	528	0	1

For the fracture-type-specific number of patients, the number of neck fractures increased over time until 2018 in men, but it did not increase in women from 2013 (Table 1). There was no increase in the number of trochanteric fractures from 2011 for men or from 2013 for women. For neck fractures, the age group–specific number of patients during the survey period was highest in those aged 85–89 years followed by those aged 80–84 years in both men and women (Supplement 1). For trochanteric fractures, the number was highest in those aged 85–89 years, followed by those aged 80–84 years in men. In women, the number was the highest in those aged 90 years or older, followed by those aged 85–89 years.

### Age- and sex-specific incidence of hip fractures

**Fig. 1:**
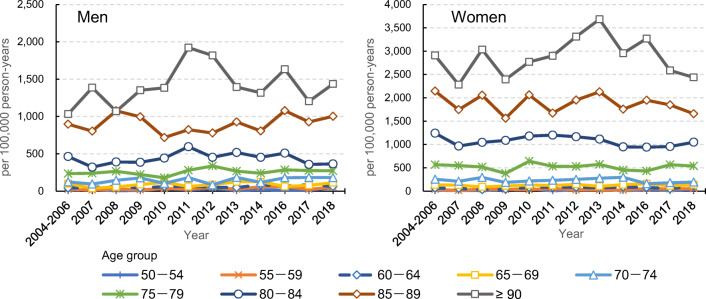
Age- and sex-specific incidence rates of hip fracture in Tottori Prefecture, Japan, from 2007 to 2018. Data are incidence rates per 100,000 person-years. Incidence rates from 2004 to 2006, which were previously reported [4], were used for comparison. (Refer actual data in Supplement 2 although the incidence rates for the age groups from 50 to 74 are too low to distinguish)

Similar to our report on the period up to 2006, age- and sex-specific incidence was low in those under 70 years and began to rise from age 70 years, rising exponentially with age thereafter. For men, the incidence rate during the survey period (per 100,000 person-years) ranged from 718.4 (2010) to 1076.9 (2016) for those aged 85–89 years and from 1069.2 (2008) to 1922.2 (2011) for those aged 90 years or older (Fig. 1, Supplement 2). For women, the incidence rate ranged from 1561.2 (2009) to 2126.5 (2013) and 2283.0 (2007) to 3683.4 (2013), respectively.

### Age-, sex-, and fracture-type-specific incidence of hip fractures

**Fig. 2:**
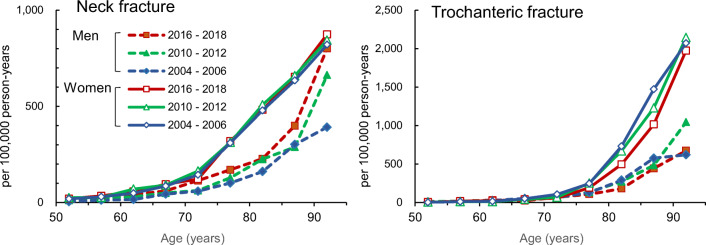
Age-, sex-, and fracture-type-specific incidence rates from 2010 and 2018. Data are mean incidence rates per 100,000 person-years for three years with 3-year intervals. Incidence rates from 2004 to 2006, which were previously reported [4], were used for comparison

During the survey period (2010–2018), the incidence rate of neck fractures (per 100,000 person-years) rose exponentially with age from 80 years of age in men. Three-year averages were 341.5 between 2007 and 2009, 662.6 between 2010 and 2012, and 883.9 between 2016 and 2018 for men aged 90 years or older (Fig. 2, Supplement 3). For women, the incidence rate rose linearly with age from 70 years of age. Three-year averages were 688.9 between 2007 and 2009, 843.7 between 2010 and 2012, and 883.2 between 2016 and 2018 for women aged 90 years or older.

The incidence rate of trochanteric fractures was higher in those aged 80 years or older compared with neck fractures, and trochanteric fractures were more frequent in older adults (Fig. 2, Supplement 3). For both men and women, the incidence rate of trochanteric fractures (per 100,000 person-years) increased with age from their late 70s. Three-year averages for men aged 90 years or older were 909.1 between 2007 and 2009, 1044.8 between 2010 and 2012, and 753.5 between 2016 and 2018. Three-year averages for women were 1876.6 between 2007 and 2009, 2144.5 between 2010 and 2012, and 2179.1 between 2016 and 2018 for women aged 90 years or older.

The trochanteric fracture incidence rate increased more than the neck fracture incidence rate with increasing age, and the neck fracture to trochanteric fracture ratio (neck/troch) was ≥ 1.0 for age 45–79 years but ≤ 1.0 for age 80 years and older in both men and women (Supplement 3).

### Estimated number of patients with hip fractures in Japan

Calculations based on the mean sex- and age group–specific incidence rates for the 3-year period from 2016 to 2018 gave an estimate of 215,091 hip fracture cases (55,550 men; 159,541 women) in those aged 35 years and older for all of Japan. Based on this incidence rate and the projected future population of Japan that was published in 2017 [2], the estimated future number of hip fracture patients in Japan is 276,879 (70,481 men; 206,397 women) in 2030 and 304,025 (76,894 men; 227,131 women) in 2040. Of these, the proportion of age 90 years or older patients, which was 24.7% in 2018, would reach 31.4% in 2030 and 41.3% in 2040.

### Changes with time in age- and sex-specific incidence rates

Compared with the period from 2004 to 2006, the sex- and age group–specific incidence rates changed differently by age group in men. The incidence rate peaked in 2012 for those aged 75–79 years and in 2011 for those aged 80–84 years and those aged 90 years and older, after which the rate tended to decrease over time, but no decrease in incidence rate over time was observed in those aged 85–89 years (Fig. 1, Supplement 2). For women, the incidence rate rose over time in all age groups but peaked in 2013 in age groups of 75 years and over, except for those aged 80–84 years, showing a peak in 2011.

**Fig. 3:**
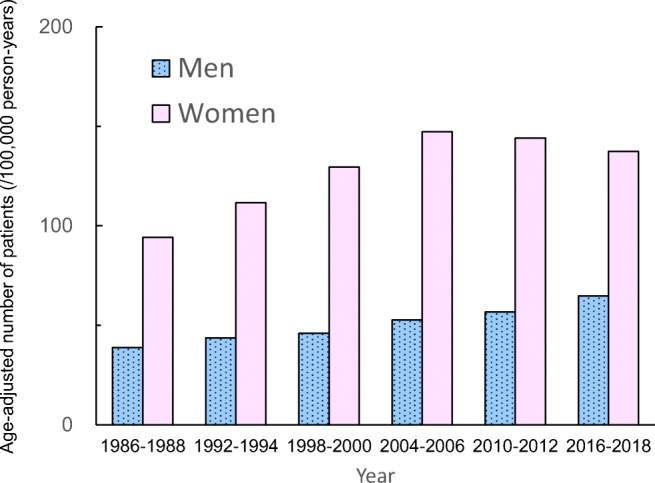
The expected number of patients age-adjusted to the population structure. The expected number of patients (per 100,000 person-years) age-adjusted to the population structure in all of Japan in 1986 (35 years and older) was calculated every 3 years with 3-year intervals. Data until 2006, which were previously reported [3–5], were used for comparison

The age-adjusted number of patients (per 100,000 person-years) adjusted by demographic structure in all of Japan in 1986 (35 years and older) based on the mean incidence rate for each 3-year period from 1986 showed an increase in incidence over time to 2016–2018 compared with the incidence for 1986–1988 in men (*p* < 0.001) (Fig. 3). In women, the incidence rose over time compared with the incidence for 1986–1988 until 2004–2006 (*p* < 0.001), and no further increase was observed from 2010.

Investigation of the age- and sex-specific incidence rates for each type of fracture showed higher age-specific incidence rates of neck fracture in men in 2010–2012 and 2016–2018 compared with 2004–2006, and a significant increase over the survey period (Fig. 2) (*p* < 0.001). The increase was especially pronounced in those aged 85–89 years and those aged 90 years and older. In women, there were no changes in age-specific incidence rates during the observation period and no differences from the 2004–2006 survey data. The age-specific incidence rates of trochanteric fracture did not change over time compared with 2004–2006 during the observation period in men. For women, among those aged 80–84 years and those aged 85–89 years, the incidence rates were lower than those in 2004–2006, but no significant changes over time were observed.

### Location and month of injury

During the study period, fractures occurred indoors in 1644 men (66.5%) and 7277 women (80.2%) (location of injury unknown for 112 men and 521 women). The proportion of fractures occurring indoors in those aged 90 years or older was 81.8% for men and 89.0% for women.

**Fig. 4:**
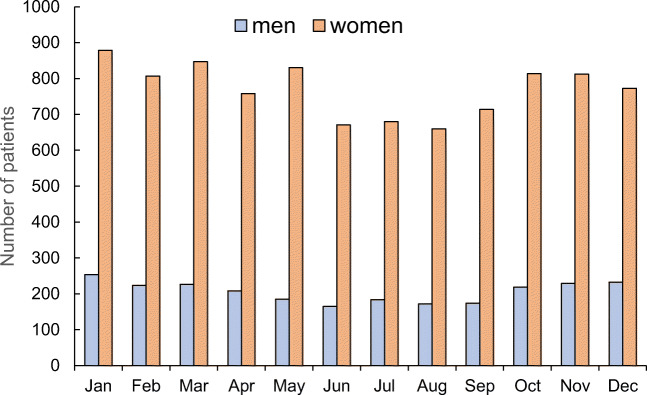
Monthly variations in the number of patients with hip fractures. Data are total number of patients during the observational period (from 2007 to 2018)

The total number of patients by month of injury during the survey period was the highest in January, with 1133 patients, followed by March with 1073 patients and November with 1041 patients (Fig. 4). It was the lowest in August, with 832 patients, followed by June, with 836. The number of patients by month of injury varied significantly (*p* < 0.001), with more fractures occurring during the winter than the summer.

## Discussion

The prevalence of osteoporosis varies with diagnostic standards and evaluation methods, hindering comparison of osteoporosis prevalence among different populations and observation of temporal changes. The incidence and prevalence rates of fragility fractures are used as indicators of osteoporosis. Vertebral fractures, which are the most commonly occurring type of fragility fracture, have no or mild symptoms and are not diagnosed by a medical institution in two-thirds of cases [6, 7]. In contrast, hip fractures result in pain and most cases require surgical treatment. Therefore, the hip fracture incidence rate is used as an indicator of osteoporosis, and regional and racial differences, as well as changes over time within regions, have been investigated. Sex- and age-specific incidence rates of hip fracture during the observation period of the present study tended to continue increasing in men, but no increase was observed after 2010 in women. Divided by type of fracture, the incidence rate of neck fractures increased over time in men, but did not change over time in women, whereas the incidence rates of trochanteric fractures did not differ from the 2004–2006 survey results for either men or women. The present study was conducted over many years from 1986 in the same area using the same methods. It is the first to show the changes in the incidence rates of all hip fractures and of each type of fracture over more than 30 years in Japan. The incidence of each type of fracture over the long term, which was first shown by the present study, is an important finding in terms of both clinical and healthcare economics, since neck and trochanteric fractures have different relationships with osteoporosis and completely different orthopedic procedures [1, 8].

Since the same methods were used in this study and our previous study, we were able to observe changes in incidence rates over a long period. The reason we were able to conduct this study over more than 30 years from 1986 was that Tottori Prefecture faces the Sea of Japan and is surrounded by mountains, so that residents who sustained a fracture while living at home could not receive medical care outside the prefecture, giving us information about all such cases. Another reason was that we collaborated with all hospitals that treat hip fractures in Tottori Prefecture, since they are closely related to the Orthopedic Department of Tottori University, allowing us to conduct accurate surveys. A third reason was that the prefecture has a high proportion of older adults, resulting in a high frequency of hip fractures, which occur commonly in older adults. The proportion of the population 65 years and older was 31.3% in Tottori and 28.1% in all of Japan, in 2018, whereas it was 14.1% and 10.6%, respectively, in 1986; thus, the aging rate increased drastically across Japan. In Tottori prefecture, the average disposable income per month was US$3959 per family unit (US$4042 for all of Japan) in 2018 [9], and the average consultation rate to medical doctors for all of Japan was 5675 (/100,000 person) in 2017, whereas that in Tottori Prefecture was 5638, the same as the average for all of Japan [10]. Based on these data, Tottori Prefecture is representative of Japan in terms of the family economics and medical resources.

The sex- and age group–specific incidence rates showed a peak in men aged 80–84 years and 90 years and older in 2011, after which the rates did not increase, although the rate did tend to increase in those aged 85–89 years, which was the largest group. In both men and women, the population aged 85 years or older is smaller than each of the under 85-year-old populations, resulting in variation by survey year. We then calculated the age-adjusted number of patients (per 100,000 person-years) for 3-year intervals based on sex- and age group–specific incidence rates and found a temporal increase in the incidence rate compared with 1986–1988 in men, but a decrease in the incidence rate from 2010 in women. Including our own study, three continuous long-term studies on hip fracture incidence rates have been conducted in Japan to date. In Niigata Prefecture, a study was conducted from 1985 to 2015, in which sex- and age-specific incidence rates were lower in 2015 than in 2010 [11]. In a sampling survey of hip fracture treatment centers in Japan [12], in those aged 60–79 years, a decrease was observed in sex- and age-specific incidence rates from 1992 to 2012 in both men and women. However, no decrease was observed in those aged 80 years or older, and the incidence for men aged 90 years or older was higher in 2012 than in 2007. Recently, Tamaki et al. used the Japanese National Database of Health Insurance Claims to calculate hip fracture incidence rates and found an increase over time in men, but not in women during the study period from 2012 to 2015 [13]. In men, the incidence rate increased over time in those aged 85 years or older, but not in those aged 70–84 years. The sex- and age-specific number of hip fractures for the 488,759 patients registered in Japan between 2009 and 2014 showed a decrease in incidence in those aged 75–79 years in both men and women during the study period [14]. These recent findings from Japan are very consistent with the results of the present study, showing a decrease in incidence rate in women from 2010 and no increase in incidence rates over time in men under 85 years of age.

In Europe, North America, and Oceania, hip fracture incidence rates increased up until the mid-1990s, after which they decreased, especially in women [15, 16]. In Asia, a study of hip fracture incidence rates over time in Beijing, China, showed a 1.61-fold increase in men and a 2.76-fold increase in women from 1990 to 2006 [17]. Meanwhile, in Taiwan, the age-standardized hip fracture incidence decreased by 13.4% in women and 12.2% in men from 2004 to 2011 [18]. Compared with the results in 2010, the crude hip fracture incidence in 2015 in Tangshan, China, increased in women, but decreased slightly in men [19]. In Hong Kong, the hip fracture incidence rate, which more than doubled from the 1960s to the 1980s, decreased in both men and women from 1995 to 2004 [20]. In a systematic review of the studies in South Korea from 2000, no temporal changes were observed in incidence rates for either men or women [21]. These findings show that, except for mainland China, hip fracture incidence rates have not been increasing in the different parts of Asia in recent years, and they have been decreasing in some places, similar to the results of the present study.

There are some hypotheses to explain the recent decrease in hip fracture incidence. Protection against fractures by osteoporosis treatment may be one factor causing the decrease in the hip fracture incidence rate. The hip fracture incidence rate was observed to decrease with the start of bisphosphonate use [22], and the impact of pharmacotherapy on fracture incidence was estimated to be as high as 40% [22]. Alendronate, a type of bisphosphonate that has effects on fragility fractures including hip fracture prevention, was approved as an osteoporosis treatment in 2001 in Japan, 7 years after alendronate treatment began in Western countries. The period of delay in the start of treatment with this bisphosphonate nearly matches the period from when the hip fracture incidence rate stopped increasing in Western countries and when it stopped increasing in Japan. Although the pharmaceutical treatment rate in patients with osteoporosis in Japan is not available, it would be lower than the mean in the EU27 [23], but it has been increasing recently (personal communication). It has been reported that compliance (medication possession ratio, MPR) over 1 year was 70.6% and 77.7% in patients newly prescribed weekly or monthly bisphosphonate therapy, respectively [24], and it was 77.5 % in patients treated with raloxifene [25] among Japanese patients with postmenopausal osteoporosis. Birth cohort effects may be another significant factor. The mean body mass index of Japanese people aged 85 years and older in 1973 and 2018 was 19.0 kg/m^2^ and 22.8 kg/m^2^, respectively, for men, and 19.5 kg/m^2^ and 22.3 kg/m^2^, respectively, for women, showing an improvement in physique [26]. Peak bone mass formation may be affected by calcium intake during this period, since the average calcium intake in 1950, when the 85-year-old in 2018 was growing, was 271 mg/day, increasing over time from 389 mg/day in 1960 to 536 mg/day in 1970 [26]. In a cohort comparison study in Japan, surveys in 1990 and 2000 showed a significant increase in bone mineral density in men in their 60s and women in their 50s, and the increase was attributed to an increase in calcium intake [27]. A survey by the Japanese Ministry of Education, Culture, Sports, Science and Technology found a mean increase of 2 kg in grip strength, 20 s in one-leg standing time with eyes open, and 60 m in 6-min walking distance in Japanese women aged 75–79 years from 1998 to 2018 [28], which indicates that physical performances of women aged 75–79 years old in 2018 were equivalent to those of women 12 years younger in 1998. We therefore know that physique, nutrition, bone mineral density, and physical functioning have improved over time in older adults, and these improvements may have contributed to the decrease in fracture incidence rates. In addition, various social circumstances are known to contribute to the incidence of hip fracture. Indicators of socioeconomic status, life expectancy, health status, and degree of urbanization are implicated in the differences in the incidence of hip fracture across countries around the world [15]. Hip fracture rates were highest in countries with the highest gross national income per capita, such as those of Europe and North America. Japan has experienced rapid urbanization since the 1960s. In 1970, the number of passenger cars was 7 million, which increased to 23 million in 1980, and then to 51 million in 2000 [29]. Then, in 2010, the number of passenger cars increased to 58 million, only a small increase since 2000. Gross national income per capita in Japan continued to rise until 1990, but it has been virtually flat since the 1990s. This socioeconomic environment may be related to the trend in hip fracture incidence, but how changes in the socioeconomic environment contribute to the hip fracture risk is unknown.

The neck/troch ratio differs between Asians and Caucasians, being lower in Asians. In women aged 85 years and older, the rate was 1.55 for Norwegians [30], 1.45 for Finnish people [31], and 0.47 for Chinese people [19], and it was 0.63 for those aged 85–89 years and 0.41 for those aged 90 years or older in the present study (mean for 2016–2018). The factor contributing to this difference may be that the hip axis length is shorter in Asians than in Caucasians. In the present study, fracture-type-specific incidence rates were examined from 1986 to 2008. Although an increase in neck fracture and trochanteric fracture incidence rates was observed in both men and women from 1986 to 2006, only the neck fracture incidence rate in men increased over time from 2010. In Japanese people, the incidence rate of neck fractures is rising over time, relatively higher than that of trochanteric fractures [14]. This may have been caused by a height increase among older adults, resulting in a rise in the neck fracture incidence rate.

In the present study, the highest number of hip fractures occurred in winter, which is consistent with past research [32]. Reasons for this may be the many layers of clothing, inadequate home safety precautions, decreased capacity for synthesizing vitamin D, outdoor conditions such as ice and slippery pavement, temperature and number of daylight hours, hypothermia and/or impairment of coordination, and impaired vision due to winter darkness, which would increase the risk of falls. However, these points have not been adequately studied.

The present study has some limitations. The first is that data collection was carried out in the same way as in our previous study, but it may not have included patients receiving treatment outside of the prefecture or at hospitals that were not surveyed in the study. However, the number of such patients is likely small. A second limitation is that the population aged 90 years or older in Tottori Prefecture was 1674 people in 1986, and it increased drastically by 2018 to 14,889 people. This rapid change in demographic structure makes it difficult to assess temporal changes in the incidence rate in those aged 90 years or older before 2004, and it might be related to previous fracture history, which is one of the most important risks for fragility fractures, in those living longer; however, no data on previous fractures were evaluated in the current study.

In conclusion, the hip fracture incidence rate in Tottori Prefecture, Japan, tended to increase until 2018 in men, but it did not increase after 2010 in women. By type of fracture, the incidence of neck fractures also increased over time from 2010 in men, but no other temporal changes were observed.

## Electronic supplementary material

ESM 1 (DOCX 60 kb)

## Data Availability

The datasets generated and analyzed during the current study are not publicly available because of professional discretion, as they were part of patients’ records, but are available as a de-identified data set from the corresponding author on reasonable request.
