# Supplementary material for: Recent trends in the incidence of hip fracture in Tottori Prefecture, Japan: changes over 32 years
Source: Arch Osteoporos. 2020 Oct 2;15(1):152. doi: 10.1007/s11657-020-00823-3 (PMC7529614; doi:10.1007/s11657-020-00823-3)
Supplement: Supplementary file 1 [file 11657_2020_823_MOESM1_ESM.docx]

Supplement 1. Age- and sex-specific number of patients with hip fracture in each year in Tottori from 2007 to 2018

	All fracture																					
	Men											Women										
Age group, y	2007	2008	2009	2010	2011	2012	2013	2014	2016	2017	2018	2007	2008	2009	2010	2011	2012	2013	2014	2016	2017	2018
35-39	2	2	3	0	2	0	0	0	1	2	0	0	4	0	0	0	0	0	0	1	1	1
40-44	4	4	4	1	1	1	1	2	1	2	3	0	1	1	1	1	3	1	0	2	0	3
45-49	1	1	0	2	3	1	1	1	3	4	5	0	2	3	2	0	2	1	1	2	4	4
50-54	5	1	2	6	3	5	2	3	2	2	7	2	3	6	10	1	5	6	2	4	3	5
55-59	4	7	4	7	6	10	5	11	11	4	9	3	11	8	7	12	5	5	4	10	8	10
60-64	9	13	9	14	17	13	11	19	14	17	12	11	6	8	19	21	21	17	20	16	10	24
65-69	6	9	15	20	10	15	24	26	15	18	25	24	17	21	25	21	27	21	30	40	27	32
70—74	15	22	26	15	26	12	27	18	26	29	32	42	57	35	40	42	45	50	55	27	32	38
75—79	32	36	30	24	38	45	35	30	36	35	35	105	99	72	121	100	98	105	80	74	96	91
80—84	29	37	38	44	60	46	54	47	54	38	38	156	172	184	201	206	200	190	160	160	161	174
85—89	30	42	44	35	43	44	55	50	69	72	75	175	216	174	241	203	246	276	235	268	279	239
≥90	26	21	27	29	40	41	33	35	49	46	63	157	218	178	225	235	292	343	289	339	332	335
Total	*163*	*195*	*202*	*197*	*249*	*233*	*248*	*242*	*281*	*269*	*304*	*675*	*806*	*690*	*892*	*842*	*944*	*1015*	*876*	*943*	*953*	*956*

	Neck fracture																					
	Men											Women										
Age group, y	2007	2008	2009	2010	2011	2012	2013	2014	2016	2017	2018	2007	2008	2009	2010	2011	2012	2013	2014	2016	2017	2018
35-39	0	1	0	0	1	0	0	0	1	0	0	0	1	0	0	0	0	0	0	1	1	1
40-44	1	2	1	0	0	0	0	1	1	1	0	0	1	1	1	1	3	1	0	1	0	2
45-49	0	0	0	1	2	0	0	1	1	2	4	0	2	2	1	0	2	0	1	2	2	4
50-54	4	0	2	2	2	2	2	2	2	1	4	2	3	6	8	1	5	5	0	3	2	5
55-59	4	1	2	5	4	4	4	6	6	2	7	3	9	7	6	9	2	5	4	5	6	8
60-64	6	1	3	7	6	9	6	14	7	12	5	9	4	5	15	18	19	16	18	12	6	17
65-69	6	1	5	10	5	10	18	14	10	16	16	20	13	15	16	14	20	18	23	23	21	25
70—74	7	3	11	4	13	10	19	13	16	18	21	23	38	21	27	33	29	38	37	16	23	28
75—79	17	17	11	11	15	26	6	14	20	21	24	55	44	35	64	56	56	67	45	41	64	58
80—84	11	14	15	21	24	23	21	21	26	24	22	70	67	84	91	88	82	85	66	80	79	84
85—89	9	19	19	16	17	12	29	27	30	34	38	62	77	67	85	65	90	94	90	108	92	103
≥90	6	5	9	10	13	20	6	13	29	24	32	45	58	45	63	67	82	113	95	87	111	92
Total	*71*	*64*	*78*	*87*	*102*	*116*	*111*	*126*	*149*	*155*	*173*	*289*	*317*	*288*	*377*	*352*	*390*	*442*	*379*	*379*	*407*	*427*

	Trochanteric fracture																					
	Men											Women										
Age group, y	2007	2008	2009	2010	2011	2012	2013	2014	2016	2017	2018	2007	2008	2009	2010	2011	2012	2013	2014	2016	2017	2018
35-39	2	1	3	0	1	0	0	0	0	2	0	0	3	0	0	0	0	0	0	0	0	0
40-44	2	2	3	1	1	1	1	1	0	1	3	0	0	0	0	0	0	0	0	1	0	1
45-49	1	0	0	1	1	1	1	0	2	2	1	0	0	1	1	0	0	1	0	0	2	0
50-54	1	1	0	4	1	3	0	1	0	1	3	0	0	0	2	0	0	1	2	1	1	0
55-59	0	6	2	2	2	6	1	5	5	2	2	0	2	1	1	3	3	0	0	5	2	2
60-64	3	12	6	7	11	4	5	5	7	5	7	2	2	3	3	3	2	1	2	4	4	7
65-69	0	8	10	9	5	5	6	12	5	2	9	4	4	6	9	7	7	3	6	17	6	7
70—74	8	19	13	11	13	2	8	5	10	11	11	18	19	13	13	9	16	12	18	11	9	10
75—79	15	19	19	13	23	19	28	15	16	14	11	50	55	37	56	44	42	38	34	33	32	33
80—84	18	22	22	23	36	23	33	24	28	13	16	85	105	100	109	118	118	105	92	79	82	89
[illegible]	[illegible]	[illegible]	[illegible]	[illegible]	[illegible]	[illegible]	[illegible]	[illegible]	[illegible]	[illegible]	[illegible]	[illegible]	[illegible]	[illegible]	[illegible]	[illegible]	[illegible]	[illegible]	[illegible]	[illegible]	[illegible]	[illegible]

Supplement 2. Age- and sex-specific incidence of hip fracture in Tottori from 2007 to 2018

Age group, y		35-39	40-44	45-49	50-54	55-59	60-64	65-69	70—74	75—79	80—84	85—89	≥90
Men	2007	11.2	24.4	5.6	23.8	15.7	49.2	37.6	95.0	239.7	320.2	802.6	1384.5
	2008	10.9	24.7	5.7	5.0	28.9	64.3	55.6	143.2	265.3	389.9	1074.2	1069.2
	2009	15.9	24.7	0.0	10.2	17.5	41.1	88.2	176.0	222.7	386.0	995.0	1351.4
	2010	0.0	6.2	11.8	31.8	31.6	59.9	117.4	103.4	179.4	442.4	718.4	1384.2
	2011	10.4	5.8	18.6	16.5	28.5	67.4	63.0	179.7	281.0	595.4	821.7	1922.2
	2012	0.0	5.7	6.2	28.5	49.2	52.7	87.0	82.8	335.7	451.7	778.5	1815.8
	2013	0.0	5.5	6.2	11.6	25.6	47.0	125.4	183.2	267.4	516.4	927.3	1393.6
	2014	0.0	10.7	6.2	18.0	57.8	86.1	125.2	116.2	238.9	453.4	805.4	1316.3
	2016	5.9	5.2	17.4	12.5	61.6	67.9	62.6	177.7	284.7	508.9	1076.9	1630.1
	2017	12.1	10.4	22.8	12.5	23.1	85.3	76.6	182.9	276.1	358.5	927.4	1202.7
	2018	0.0	16.1	27.5	44.0	53.1	62.7	112.2	181.2	271.0	364.4	1001.1	1433.8
Women	2007	0.0	0.0	0.0	9.6	11.9	57.8	128.3	211.7	546.2	965.8	1745.8	2283.0
	2008	21.9	5.9	11.0	14.9	45.5	29.1	90.7	291.9	515.7	1043.2	2053.4	3034.5
	2009	0.0	5.9	16.8	30.3	35.0	36.3	109.0	186.0	379.0	1084.0	1561.2	2392.8
	2010	0.0	6.0	11.4	51.8	31.9	80.3	132.0	217.9	640.6	1179.5	2062.5	2766.8
	2011	0.0	5.8	0.0	5.4	56.5	82.7	118.6	231.6	531.4	1198.2	1670.4	2900.2
	2012	0.0	17.0	11.9	27.3	24.3	84.1	144.2	249.9	526.4	1164.0	1952.1	3309.9
	2013	0.0	5.5	6.0	33.3	25.1	71.1	103.4	277.4	572.3	1112.2	2126.5	3683.4
	2014	0.0	0.0	6.0	11.3	20.4	88.6	138.1	297.3	450.9	946.6	1754.8	2955.3
	2016	6.0	10.4	11.5	23.9	53.9	75.6	160.4	158.3	432.4	940.9	1948.2	3267.2
	2017	6.1	0.0	22.8	18.0	43.9	48.8	109.9	177.9	564.7	956.3	1848.5	2585.4
	2018	6.3	16.4	22.1	30.2	55.8	121.3	135.9	194.2	535.9	1048.2	1657.4	2436.4

Data are per 100,000 person-years.

Supplement 3. Age-, sex- and fracture type- specific incidence of hip fracture in Tottori from 2007 to 2018

	Age group, y	35-39	40-44	45-49	50-54	55-59	60-64	65-69	70–74	75–79	80–84	85–89	≥90
	Observation year												
Neck fracture Men	2007	0.0	6.1	0.0	19.1	15.7	32.8	37.6	44.3	127.3	121.5	240.8	319.5
	2008	5.5	12.3	0.0	0.0	4.1	4.9	6.2	19.5	125.3	147.5	485.9	254.6
	2009	0.0	6.2	0.0	10.2	8.7	13.7	29.4	74.5	81.7	152.4	429.7	450.5
	2010	0.0	0.0	5.9	10.6	22.6	29.9	58.7	27.6	82.2	211.1	328.4	477.3
	2011	5.2	0.0	12.4	11.0	19.0	23.8	31.5	89.8	110.9	238.1	324.9	624.7
	2012	0.0	0.0	0.0	11.4	19.7	36.5	58.0	69.0	193.9	225.9	212.3	885.7
	2013	0.0	0.0	0.0	11.6	20.5	25.7	94.1	128.9	45.8	200.8	489.0	253.4
	2014	0.0	5.4	6.2	12.0	31.5	63.5	67.4	83.9	111.5	202.6	434.9	488.9
	2016	5.9	5.2	5.8	12.5	33.6	34.0	41.7	109.3	158.2	245.0	468.2	964.7
	2017	0.0	5.2	11.4	6.2	11.5	60.2	68.1	113.5	165.7	226.4	518.7	747.9
	2018	0.0	0.0	22.0	25.1	41.3	26.1	71.8	118.9	185.9	211.0	563.2	939.0
Women	2007	0.0	0.0	0.0	9.6	11.9	47.3	106.9	115.9	286.1	433.4	618.5	654.4
	2008	5.5	5.9	11.0	14.9	37.3	19.4	69.4	194.6	229.2	406.4	732.0	807.3
	2009	0.0	5.9	11.2	30.3	30.7	22.7	77.9	111.6	184.2	494.9	601.2	604.9
	2010	0.0	6.0	5.7	41.4	27.4	63.4	84.5	147.1	338.8	534.0	727.4	774.7
	2011	0.0	5.8	0.0	5.4	42.3	70.9	79.1	182.0	297.6	511.8	534.8	826.9
	2012	0.0	17.0	11.9	27.3	9.7	76.1	106.8	161.0	300.8	477.2	714.2	929.5
	2013	0.0	5.5	0.0	27.8	25.1	66.9	88.6	210.8	365.2	497.6	724.2	1,213.5
	2014	0.0	0.0	6.0	0.0	20.4	79.7	105.9	200.0	253.6	390.5	672.0	971.5
	2016	6.0	5.2	11.5	17.9	27.0	56.7	92.2	93.8	239.6	470.4	785.1	838.5
	2017	6.1	0.0	11.4	12.0	32.9	29.3	85.5	127.9	376.4	469.3	668.4	1,009.9
	2018	6.3	11.0	22.1	30.2	44.7	85.9	106.2	143.1	341.6	506.0	748.0	801.3
Trochanter fracture Men	2007	11.2	12.2	5.6	4.8	0.0	16.4	0.0	50.7	112.4	198.7	561.8	1,011.7
	2008	5.5	12.3	0.0	5.0	24.8	59.3	49.4	123.6	140.0	231.8	588.2	814.7
	2009	15.9	18.5	0.0	0.0	8.7	27.4	58.8	88.0	141.1	223.5	565.4	900.9
	2010	0.0	6.2	5.9	21.2	9.0	29.9	52.8	75.9	97.2	231.2	390.0	906.9
	2011	5.2	5.8	6.2	5.5	9.5	43.6	31.5	89.8	170.1	357.2	496.8	1,297.5
	2012	0.0	5.7	6.2	17.1	29.5	16.2	29.0	13.8	141.7	225.9	566.2	930.0
	2013	0.0	5.5	6.2	0.0	5.1	21.4	31.4	54.3	214.0	315.5	438.4	1,140.2
	2014	0.0	5.4	0.0	6.0	26.3	22.7	57.8	32.3	119.4	231.5	370.5	827.4
	2016	0.0	0.0	11.6	0.0	28.0	34.0	20.9	68.3	126.5	263.9	593.1	665.3
	2017	12.1	5.2	11.4	6.2	11.5	25.1	8.5	69.4	110.4	122.6	579.7	685.6
	2018	0.0	16.1	5.5	18.8	11.8	36.6	40.4	62.3	85.2	153.4	548.4	909.6
Women	2007	0.0	0.0	0.0	0.0	0.0	10.5	21.4	90.7	260.1	526.3	1,127.3	1,628.6
	2008	16.4	0.0	0.0	0.0	8.3	9.7	21.3	97.3	286.5	636.8	1,321.4	2,213.3
	2009	0.0	0.0	5.6	0.0	4.4	13.6	31.1	69.1	194.8	589.1	951.1	1,787.9
	2010	0.0	0.0	5.7	10.4	4.6	12.7	47.5	70.8	296.5	639.6	1,309.4	1,979.8
	2011	0.0	0.0	0.0	0.0	14.1	11.8	39.5	49.6	233.8	686.3	1,135.5	2,073.3
	2012	0.0	0.0	0.0	0.0	14.6	8.0	37.4	88.9	225.6	686.8	1,237.9	2,380.4
	2013	0.0	0.0	6.0	5.6	0.0	4.2	14.8	66.6	207.1	614.6	1,394.6	2,469.9
	2014	0.0	0.0	0.0	11.3	0.0	8.9	27.6	97.3	191.6	544.3	1,045.4	1,912.3
	2016	0.0	5.2	0.0	6.0	27.0	18.9	68.2	64.5	192.8	464.6	1,155.9	2,419.0
	2017	0.0	0.0	11.4	6.0	11.0	19.5	24.4	50.0	188.2	487.1	1,351.3	2,001.6
	2018	0.0	5.5	0.0	0.0	11.2	35.4	29.7	51.1	194.3	536.1	987.7	2,116.5
